# Inheritance Mode of a Red-Eye Mutation in *Macrolophus pygmaeus* (Hemiptera: Miridae)

**DOI:** 10.3390/insects16070709

**Published:** 2025-07-10

**Authors:** María del Carmen Reche, Carolina Grávalos, Virginia Balanza, Ana Belén Abelaira, Amador Rodríguez-Gómez, Pablo Bielza

**Affiliations:** Biocontrol Selection Lab, Departamento de Ingeniería Agronómica, Universidad Politécnica de Cartagena, 30203 Cartagena, Spain; mariadelcarmen.reche@edu.upct.es (M.d.C.R.);

**Keywords:** biological control agent, mutation, red eyes, autosomal recessive allele

## Abstract

A mutation affecting eye color was discovered in *Macrolophus pygmaeus* (Rambur) (Hemiptera: Miridae). This species is an important biological control agent used to manage whiteflies and other arthropod pests in greenhouse crops. Typically, these predators exhibit a dark eye coloration, ranging from garnet to black. However, individuals with bright red eyes have been identified within wild populations, making them easily distinguishable. The objective of this study was to study the mode of inheritance of the red eye color mutation observed. Additionally, a comparative analysis of key biological traits was conducted between individuals carrying the mutation and those from a wild-type population. From these specimens, a population carrying this eye color mutation, referred to as *ruby*, was established. Genetic analyses revealed that the red coloration is controlled by a single recessive allele located on an autosomal chromosome. Red-eyed individuals exhibited superior performance in biological traits, including greater body size in both males and females, as well as increased fecundity and longevity in females. The utilization of this red-eyed variant could be valuable as a visual marker in ecological and biological studies of population dynamics.

## Simple Summary

*Macrolophus pygmaeus* is an important biological control agent used to manage whiteflies and other arthropod pests in greenhouse crops. The typical eye color of adult individuals of this predator ranges from garnet to black. However, adults with bright red eyes were found in wild populations. The aim of this study was to determine the mode of inheritance of this mutation. In addition, the biological traits of the red-eyed population were compared with those of a normal population. To achieve this, a laboratory population carrying this red eye color mutation was established. The mutation, known as *ruby*, is controlled by a single autosomal recessive allele. The red-eyed individuals showed superior performance in certain traits, such as the size of both males and females, as well as the fecundity and longevity of females. Finally, it is suggested that this red-eyed variant could serve as a useful visual marker for scientific studies on the biology and ecology of this beneficial insect.

## 1. Introduction

*Macrolophus pygmaeus* (Rambur) (Hemiptera: Miridae) is a natural enemy with a key role in the biological control of whiteflies and other small arthropod pests in protected crops in the temperate and Mediterranean regions of Europe [1,2,3]. With the aim of enhancing its performance in the field and optimizing its large-scale rearing in biofactories, *M. pygmaeus* has been the subject of numerous studies in various laboratories to deepen the understanding of its biology and ecology [4,5,6].

**Figure 1:**
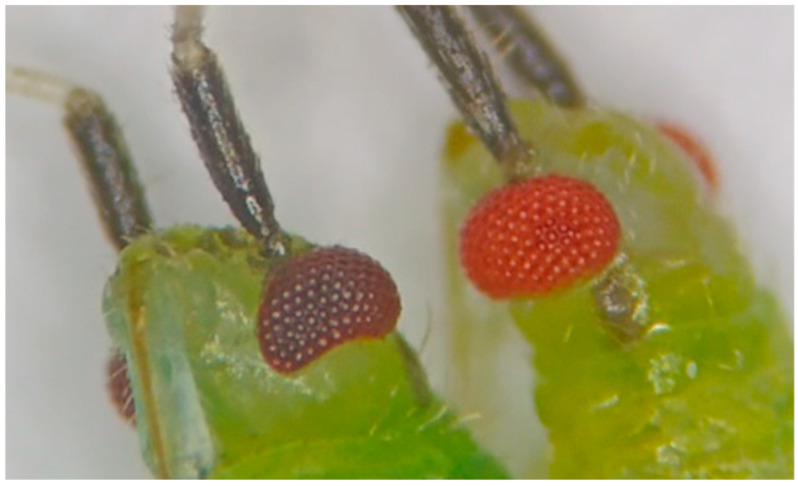
Detail of the eye of *Macrolophus pygmaeus*. (**Left**): Normal strain. (**Right**): Red-eyed strain.

In 2022, a single female *M. pygmaeus* with bright red eyes (Figure 1) was observed. This female was used to establish a population of red-eyed *M. pygmaeus*. In insects, most mutations that alter eye color are recessive and usually involve a single gene [7]. No previous studies have documented populations of *M. pygmaeus* with bright red eyes. However, similar studies have been conducted on *Orius sauteri* and *O. strigicollis* (Heteroptera: Anthocoridae) and *Lygus lineolaris* (Hemiptera: Miridae) [8,9]. In both studies, it was concluded that the mutant red eye color was determined by a single autosomal recessive allele.

Eye-color mutants have been exploited for over a century as convenient, scorable markers in insects because a single recessive allele can produce a conspicuous phenotype without impairing viability. In *Drosophila melanogaster*, more than 85 eye-color loci have been described, which fall into three functional classes: (i) enzymes of the ommochrome or pteridine biosynthetic pathways (e.g., *vermilion*, *cinnabar*), (ii) ATP-binding-cassette transporters that import precursors into lysosome-derived pigment granules (e.g., *white*, *scarlet*, *brown*), and (iii) the so-called “granule-group” genes that control biogenesis and cargo delivery to those granules [10].

Granule-group mutations illustrate how defects in vesicle trafficking can mimic the loss of pigment enzymes. Classical alleles of *ruby*, *garnet*, *carmine,* and *orange* disrupt the four sub-units of the adaptor-protein-3 (AP-3) complex; as a consequence, both brown ommochromes and red pteridines fail to accumulate, yielding brick-red eyes and, in stronger alleles, reduced adult fitness [11]. More recent forward-genetic screens have uncovered additional trafficking genes whose loss has similar effects, including the vacuolar ATPase sub-unit (*chocolate*) and components of the HOPS/CORVET tethering complex (*deep-orange*, *carnation*) [11].

Two studies emphasize that single-gene lesions outside the classical pathways are sufficient to alter insect eye color. A missense mutation in the t-SNARE gene *SNAP29* was shown to be the long-sought *purpleoid 1* allele: homozygous flies display viable but maroon-colored eyes because defective SNARE pairing blocks pigment-granule fusion [12]. Likewise, an RNase-dead allele of the ER-stress sensor *Ire1* abolishes pigment granules altogether, leading to a marked reduction of both ommochrome and pteridine pigments and secondary changes in photoreceptor physiology [13]. Orthologues of AP-3 sub-units, HOPS tethers, SNAREs, and *Ire1* are conserved in hemipterans and other non-dipteran insects, suggesting that mutations in these genes can elicit red-eye phenotypes well beyond *Drosophila*.

Given this broad genetic landscape, the aim of the present study was to compare life-history traits of bright-red-eyed *M. pygmaeus* with those of wild-type and hybrid lines, and to determine whether the red-eyed phenotype segregates as a recessive allele at an autosomal locus or might instead reflect alternative defects in pigment-granule trafficking genes.

## 2. Materials and Methods

### 2.1. Insects

The normal and red-eyed populations alike were reared in the laboratory by using 1 L plastic containers covered with filter paper on the lid, with ad libitum access to *Ephestia kuehniella* (Lepidoptera: Pyralidae) eggs as food, green bean pods (*Phaseolus vulgaris* L.) as the moisture source and egg-laying substrate, and pieces of paper as hiding places to avoid cannibalism. The populations were maintained under controlled conditions at 26 ± 1 °C, 65 ± 5% RH, and an L16:D8 light regime. The populations were reproduced in the laboratory for 2–4 generations before the experiments.

### 2.2. Development of the Red-Eyed Population

In 2022, a female exhibiting a distinct red eye color was identified within a population maintained in the Biocontrol Selection Lab (Universidad Politecnica de Cartagena, Cartagena, Spain). For the purpose of establishing a red eye mutant population (hereafter red-eyed), the red-eye-color virgin female was crossed with a normal male of *M. pygmaeus* from a standard population (hereafter Normal) established in our lab. The offspring born from them were all normal adults. These normal F1 *M. pygmaeus* progeny were interbred with each other and produced both normal and red-eyed adults. The red-eyed adults (females and males) were selected to establish a pure red-eyed population carrying the mutation *ruby*.

### 2.3. Genetic Cross

Genetic crosses were performed to analyze the inheritance mechanism of the *ruby* mutation associated with red eye pigmentation in *M. pygmaeus*. Fifth instar nymphs were isolated and reared individually until adulthood. To produce the F1 generation, virgin red-eyed females and males were mated with virgin females and males from the normal population. A total of 22 crosses were made, 9 pairs of red-eyed females and normal males and another 13 pairings of opposite sexes. The total number of nymphs was recorded and categorized by group and by the corresponding female (1 to 13).

Red eye coloration was absent in all adults resulting from the initial crosses. Upon reaching adulthood, the offspring were backcrossed with both parental lines (red-eyed and Normal). For every cross, approximately 7 to 10 virgin females, along with an equal number of virgin males, were placed into 60 mL cups with *Ephestia* eggs as food and a piece of green bean pod for oviposition. Every three days, the bean pods were exchanged for new ones, and the nymphs born were counted. Individuals were kept separately in individual containers, and their eye color was noted upon reaching adulthood. The leftover F1 males and females, following the backcrosses, were mated among themselves to produce the F2 generation. These cross experiments were maintained under controlled conditions at 26 ± 1 °C, 65 ± 5% RH, and L16:D8 light regime. The last instar nymphs were placed individually into plastic cups (30 mL) with *E. kuehniella* eggs ad libitum as food and a piece of green bean pod to prevent mating upon adult emergence. Newly emerged adults (<24 h) were sexed.

### 2.4. Nymphal Survival, Developmental and Body Weight Adult

Green bean pods were introduced in the rearing containers for the females to lay eggs. After 24 h, the bean pods were collected and these eggs were developed. Seventy-five first instars (<24 h old) were placed in an individual plastic cup (500 mL) covered with paper secured by a rubber band. For each population, 5 replicates were established, containing the bean pod provided as a water source and *E. kuehniella* eggs as food. The maintenance of these repetitions was carried out twice a week, changing the bean and adding more food. Upon adult emergence, the survivors of each population were counted. The experiment was performed under standard laboratory conditions, 26 ± 1 °C, 65 ± 5% RH, and a light regime of L16:D8. Once the adults emerged, a group of 10 males and another group of 10 females per replication were formed to study their size, with individuals being weighed on a precision scale.

### 2.5. Female Fertility and Longevity Trial

We conducted a separate experiment to study the reproductive biological characteristics of the new red-eyed population compared to a standard one. Once the nymphs from both populations emerged as adults, they were given 5 days for copulation to take place between males and females. After this period, for each population, 30 females were individually deposited in 60 mL plastic cups covered with a perforated lid to allow ventilation containing *E. kuehniella* eggs ad libitum and a piece of green bean pod.

*Macrolophus pygmaeus* eggs are not easily seen with a stereoscopic magnifying glass. Therefore, we counted the emergence of nymphs N1. The beans that were removed were placed in plastic cups (60 mL) with a small number of *E. kuehniella* eggs, and after 10 days, the number of individuals was counted. The trial was performed under standard laboratory conditions, 26 ± 1 °C, 65 ± 5% R.H. and a 16:8 (L:D) h. Every 2–3 days, food was added, and the bean was exchanged for a fresh piece. Early fertility (14 days) was assessed.

### 2.6. Data Analysis

A chi-square test was conducted to compare the observed eye color proportions (red vs. normal) with the expected ratios of 1:3 for F2 crosses and 1:1 for backcrosses. Differences in biological and ecological traits between the two populations were evaluated using one-way analysis of variance (ANOVA). Prior to analysis, assumptions of normality and homogeneity of variances were verified. When significant differences were detected, means were compared using Tukey’s honestly significant difference (HSD) test.

## 3. Results

### 3.1. Genetic Cross

The results of the red- and normal-eyed crosses comprised offspring with standard-color eyes (F1), except for those crosses when both the male and the female had red eyes (Table 1). Inbreeding between F1 individuals (RN × RN and NR × NR) produced F2 offspring, presenting the expected ratio 1:3. The same happened in the case of backcrosses using parents, for which the offspring fitted with the expected ratio 1:1.

### 3.2. Nymphal Survival and Developmental

**Figure 2:**
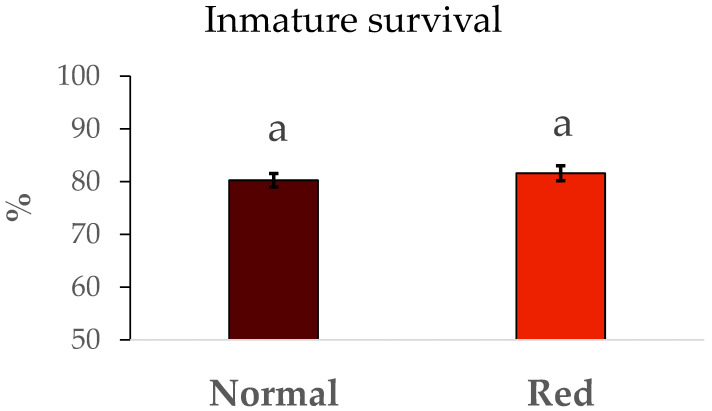
Mean survival ± SE during the development. Survival (%) from N1 to adult by population. Means within a graph followed by the same letter are not significantly different (*p* > 0.05; Tukey test). Five repetitions of 75 N1 individuals per population.

**Figure 3:**
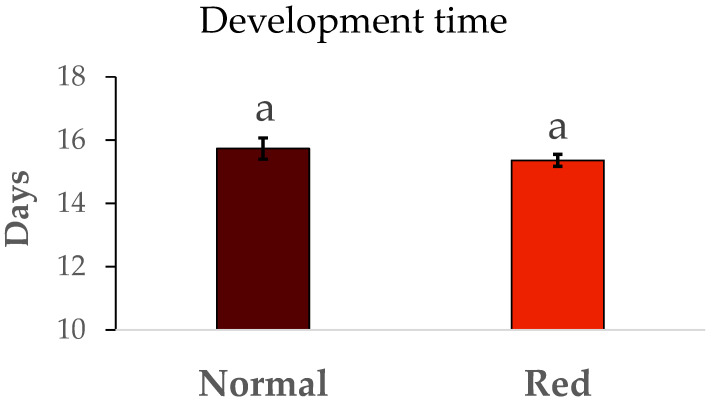
Mean development time ± SE. Development time (days) from N1 to adult by population. Means within a graph followed by the same letter are not significantly different (*p* > 0.05; Tukey test). Five repetitions of 75 N1 individuals per population.

The results of survival and duration of immature development from N1 to adult are summarized in Figure 2. No significant differences were detected in immature survival between the populations tested (F = 0.48, df = 1/8, *p* > 0.05). Similarly, the development time (Figure 3) in the red-eyed population was the same in the standard population (F = 0.93, df = 1/8, *p* > 0.05).

### 3.3. Male and Female Body Weight

**Figure 4:**
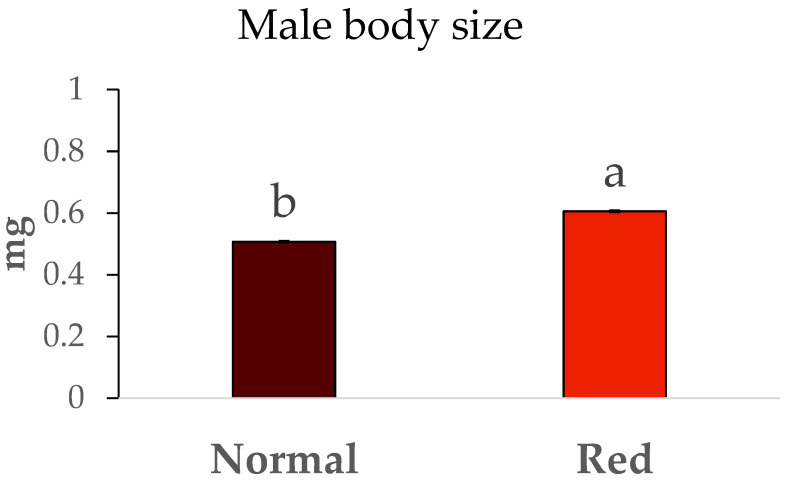
Body size (mg) ± SE of males of *Macrolophus pygmaeus* feeding on *Ephestia kuehniella* eggs. Means within a graph followed by the same letter are not significantly different (*p* > 0.05; Tukey test). A group of 10 males per replicate and population, 5 replicates per population.

**Figure 5:**
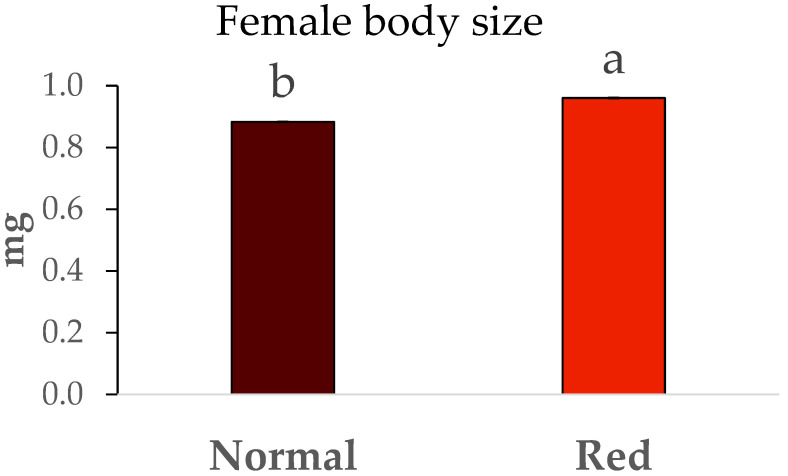
Body size (mg) ± SE of females of *Macrolophus pygmaeus* feeding on *Ephestia kuehniella* eggs. Means within a graph followed by the same letter are not significantly different (*p* > 0.05; Tukey test). A group of 10 females per replicate and population, 5 replicates per population.

The weights of males and females from both studied populations are shown in Figure 4 and Figure 5. Significant differences were observed in the sizes of males between the two populations (F = 106.71, df = 1/7, *p* < 0.05), with the males from the red-eyed population being larger, exhibiting an average weight close to 0.6 mg. In contrast, the weights of males from the standard population were around 0.5 mg. The same trend was observed with the weights of females from both populations, as significant differences were also present (F = 6.62, df = 1/7, *p* < 0.05). Once again, the females from the red-eyed population were heavier (close to 1 mg) compared to those from the normal population (approximately 0.8 mg).

### 3.4. Female Fertility and Longevity

**Figure 6:**
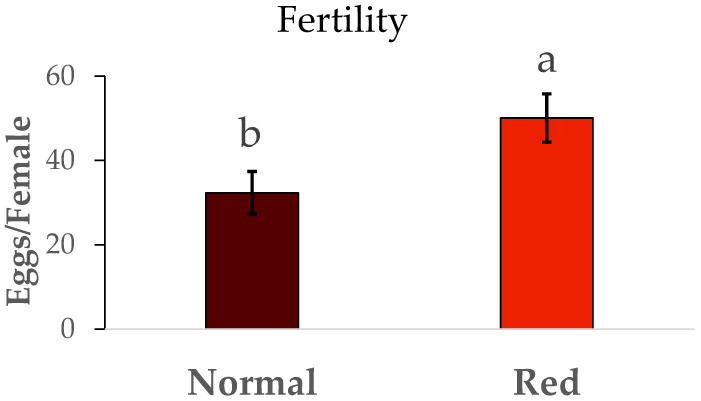
Mean total fertility (N1/female) ± SE of females of *Macrolophus pygmaeus* feeding on *Ephestia kuehniella* eggs. Means within a graph followed by the same letter are not significantly different (*p* > 0.05; Tukey test). Thirty females per population.

**Figure 7:**
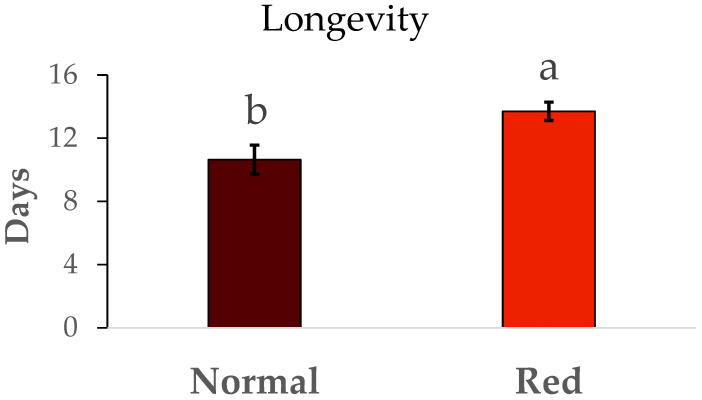
Mean longevity (days) ± SE of females of *Macrolophus pygmaeus* feeding on *Ephestia kuehniella* eggs. Means within a graph followed by the same letter are not significantly different (*p* > 0.05; Tukey test). Thirty females per population.

The results for the fertility and longevity of females from the red-eyed and normal populations are show in Figure 6. The fertility (N1/female) was much lower in the normal population than in the red-eyed population (F = 5.01, df = 1/42, *p* < 0.05). In the same way, the longevity (Figure 7) in the red-eyed population was greater than in the normal population (F = 0.93, df = 1/8, *p* < 0.05).

## 4. Discussion

Experimental crosses revealed that the *ruby* mutant of *M. pygmaeus* was controlled by a single autosomal recessive allele. Eye color mutations have been studied in other insects of the Hemiptera family, such as *Lygus lineolaris* (Hemiptera: Miridae), whose mutations affecting eye color were inherited as autosomal recessive genes [8]. There is also an orange-eyed mutant of *Nilaparvata lugens* (Hemiptera: Delphacidae) whose inheritance is controlled by a single autosomal recessive allele [14]. Mutants with red eyes were also found in the flower bugs *Orius sauteri* (Heteroptera: Anthocoridae) and *O. strigicollis* [9]. These mutants have bright red-colored eyes and are easy to differentiate. This study also demonstrated that the red-eye trait was recessive and inferred that the locus was autosomal in both species. Moreover, in another study by our research team, a mutant of *Orius laevigatus* emerged whose nymphs exhibited an orange coloration. It was also demonstrated that the inheritance of this color was controlled by a single autosomal recessive allele [15].

On the other hand, we found a study on *O. strigicollis* in which the authors demonstrated a method to obtain red-eyed offspring using a simple and accessible genetic editing technique for insects called direct parental CRISPR (DIPA-CRISPR) [16]. They focused on the *cinnabar* gene, which encodes kynurenine 3-monooxygenase, an enzyme involved in the biosynthesis of the ommochrome pigment [17,18]. The inactivation of the *cinnabar* gene results in red-eyed phenotypes in various insect species, including hemipterans [19,20]. Nevertheless, red-eyed phenotypes can also emerge from defects in genes that govern the biogenesis or trafficking of pigment granules. In *Drosophila*, the loss of function of the ER-stress sensor *Ire1* eliminates pigment granules and simultaneously reduces both ommochrome and pteridine pigments, yielding pale red eyes [13]. Missense or null alleles of the t-SNARE gene *SNAP29* (*purpleoid*) disrupt vesicle fusion and create a maroon-red eye color [12]. Likewise, mutations in the AP-3 adaptor-complex genes (*ruby*, *garnet*, *carmine*, *orange*) and other “granule-group” trafficking genes (*deep orange*, *carnation*, *light*) diminish pigment deposition and generate brick-red eyes [10,11]. These studies indicate that multiple molecular routes can converge on a red-eyed phenotype. Consequently, while the spontaneous red-eyed strain of *M. pygmaeus* is most parsimoniously explained by a naturally occurring loss-of-function in *cinnabar*—analogous to the classical *ruby* allele in flies—alternative mutations affecting pigment-granule trafficking remain plausible until molecular confirmation is obtained.

Regarding survival, previous studies have reported nymphal survival rates of *M. pygmaeus* feeding on *E. kuehniella* ranging from 80% to 90% [21,22,23]. Our findings fell within this range, at around 80% survival. Moreover, the development time we observed (approximately 16 days) aligns with the results in [22], although other authors have reported a slightly longer period (18–19 days) [20,22]. Borges et al. [23] reported a male size of *M. pygmaeus* fed on *Ephestia* eggs of 0.65 mg and females with a size of 1.1 mg. These results support those obtained for the red-eyed population; however, the weights of both sexes in the standard population were slightly lower than those of the red-eyed population. Similarly, body size plays a crucial role in a predator, as it is closely linked to the type and size of prey it can hunt [24].

Differences in fertility were observed between the two populations. Females from the standard population laid approximately 32 N1 nymphs, consistent with the findings of Vanderkerkhove et al. [21], who reported an average of 32 eggs per female from a normal eye population. However, the red-eyed population exhibited higher fertility (50 N1/female) compared to our standard population. Fertility rates reported in other studies range from 45 to 80 N1 per female from a normal eye population [22,23]. There were also differences in female longevity between the standard and red-eyed populations. However, previous studies [23] reported longer longevity from a normal eye population, ranging from 48 to 50 days. While our population’s female longevity was between 10 and 14 days.

The eye color mutation in *M. pygmaeus* serves as a highly useful visual marker for biological and ecological research on this predator used in biological control. The red-eyed population, which carries the *ruby* mutation, appears to exhibit improved performance in certain biological traits, such as body size, longevity and fecundity. For this reason, this mutation could be introgressed into high-performance strains. Strains with and without the *ruby* mutation can be used to compare the establishment and efficacy of biological control in different populations of this insect. Furthermore, the red-eyed population is valuable for studies on the dispersal and distribution of *M. pygmaeus* within and between plants, crops, and fields, preventing confusion between released individuals and wild populations. Understanding the movement and population dynamics of this predator is crucial for enhancing integrated pest management programs, particularly in augmentative and conservation biological control strategies.

In laboratory studies, the *ruby* mutation can also be employed in research on parentage and reproductive behavior, allowing the assessment of sexual competition among different strains. In conclusion, this mutation provides new opportunities for the technical and scientific advancement of *M. pygmaeus* as a biological control agent.

## Figures and Tables

**Table 1:** Adult eyes color in offspring of crosses and backcrosses between mutant (red-eyed) and standard (normal) *Macrolophus pygmaeus* populations.

Cross (Female × Male)	Red (R)	Normal (N)	Expected Ratio (R:N)	Observed Ratio (R:N)	χ^2^	*p*
R × N (9)	0	127	0:1	0:1		
N × R (13)	0	127	0:1	0:1		
R × R (12)	106	0	1:0	1:0		
RN × RN (8)	50	150	1:3	1:3.00	0.007	0.935
NR × NR (8)	50	128	1:3	1:2.56	0.749	0.387
RN × RR (8)	67	84	1:1	1:1.25	1.695	0.193
RR × RN (7)	59	76	1:1	1:1.31	1.896	0.169
NR × RR (8)	65	87	1:1	1:1.33	2.901	0.089
RR × NR (10)	84	104	1:1	1:1.24	1.920	0.166

The number in brackets is the number of couples used in each cross. The offspring of each cross was recorded in the adult stage. NR: Offspring of standard (normal) females and mutant (red-eyed) males; RN: offspring of mutant (red-eyed) females and standard (normal) males. RR: offspring of mutant (red-eyed) females and mutant (red) males.

## Data Availability

The original contributions presented in this study are included in the article. Further inquiries can be directed to the corresponding author.
